# The Influence of Community Members on Participation by Youth in an HIV Vaccine Trial in Tanzania

**DOI:** 10.1371/journal.pone.0168660

**Published:** 2016-12-20

**Authors:** Theodora Mbunda, Edith A. M. Tarimo, Guerino Chalamilla, Muhammad Bakari, Eric Sandström, Asli Kulane

**Affiliations:** 1 Department of Internal Medicine, School of Medicine, Muhimbili University of Health and Allied Sciences, Dar es Salaam, Tanzania; 2 Equity and Health Policy Research Group, Department of Public Health Sciences, Karolinska Institutet, Stockholm, Sweden; 3 Management and Development for Health, Dar es Salaam, Tanzania; 4 Department of Nursing Management, School of Nursing, Muhimbili University of Health and Allied Sciences, Dar es Salaam, Tanzania; 5 Department of Health, Dar es Salaam City Council, Dar es Salaam, Tanzania; 6 Department of Clinical Science and Education, Södersjukhuset, Karolinska Institutet, Stockholm, Sweden; Universidade Estadual de Maringa, BRAZIL

## Abstract

In sub-Saharan Africa, the burden of HIV is high among young people and it is of the utmost importance that they be recruited into vaccination trials. Since community members influence the willingness of young people to participate in the vaccination trials, ascertaining their opinions is essential to overcoming barriers to such participation. Here, in seven focus group discussions we explored the views of 44 community members identified as someone they felt close by youth in Tanzania. The transcripts of these discussions were examined using content analysis. Our participants expressed that community members would be directly involved in the decisions of young people about whether or not to participate in an HIV vaccine trial. In general, they felt that community members would provide social support for youth during the trial and perceived that youth might have misconceptions concerning the vaccine and trial process. The participants pointed out structural factors such as substance use, poverty, stigma and unemployment that are barriers to participation. In conclusion, involvement of community members could be an integral part of the recruitment and retention of young people in HIV vaccine trials in Tanzania.

## Introduction

The lives of young people worldwide, and in particular young women, are strongly affected by HIV by 2013. Because stigma and discrimination remain rife in many parts of the world, this continues to deter those most at risk from seeking essential HIV services [1]. Although prevalence of HIV among young people of sub-Saharan Africa fell by 42% between 2001 and 2014, the prevalence among women remains two times as high as among men because of emerged disturbing signs of increases in sexual risk behaviors, gender inequalities and harmful gender norms [1]. By the end of 2012, HIV prevalence in East Africa was approximately 1% and 2% for males and females aged 15–24 years old, respectively [1]. The overall prevalence of HIV in Tanzania is estimated to be 5%, while corresponding values for women and men 15–24 years old are estimated to be 3% and 1% respectively [2].

The wide array of efforts to prevent HIV that have proven effective nonetheless have certain limitations. To date have these efforts focused overwhelmingly on reducing individual risk, with fewer attempts to address for example, socio-cultural, economic, political, legal and other contextual factors that influence vulnerability to HIV [3]. Furthermore, in Tanzania, efforts to prevent HIV are impeded by stigma, risky sexual behavior, gender inequality, and discrimination [4].

In its effort to curb the spread of this disease, the Joint United Nations Programme on HIV/AIDS (UNAIDS) proposed the Investment Framework Enhanced (IFE) in 2013. This framework described projections that scaling up existing prevention, treatment, and care programs in low and middle-income countries, in combination with new approaches, including a vaccine, could reduce new infections most effectively. Accordingly, vaccination can play a critical role in eliminating HIV in a sustainable manner [5] and development of an effective, socially acceptable and affordable vaccine is needed.

Development of such a vaccine presents a major scientific challenge since HIV mutates very rapidly, allowing the virus to escape the body’ s immune responses while giving rise to numerous clades that circulate around the globe [5]. Nonetheless, a number of trials of HIV vaccines in different phases have been and are being carried out around the globe. Phases I and II focus on the vaccine’s safety and ability to elicit an immune response, while phase III evaluates protection from HIV infection [6]. The database of clinical trials of HIV vaccines maintained by the International AIDS Vaccine Initiatives shows that 222 participants have completed phase I trials, 44 phase II trials and 1 a phase III trial [7].

Tanzania has been conducting phase I and II trials of this nature since 2007. From 2010 to 2012, a phase IIa randomized clinical trial recruited 120 healthy, low risk HIV negative participants from two centers in Tanzania, Dar es Salaam and Mbeya. In Dar es Salaam these participants were police officers and prison guards, as well as young people from a youth friendly clinic, and in Mbeya the general population [8].

It is vital to enroll young people in HIV vaccination trials since their risk of contracting HIV is higher due to their perception of lower risk, low condom usage and multiple sexual partners [2]. Social Cognitive Theory explains that if an individual believes that he/she can solve a problem instrumentally; he/she becomes more inclined to do so and feels more committed to this decision [9]. This has been demonstrated in numerous studies exploring willingness to participate in biomedical HIV interventions such as vaccine trials, where people who felt compelled to take positive action to control the spread of HIV have expressed a moderate to high willingness to participate [10, 11].

In addition, an investigation among African- Americans in United States revealed that of individual and network factors that influence participation in HIV vaccine trials, social activism exerts a significant impact [12]. The respondents felt inclusion and purpose when involved in community-organized research, making them more likely to participate in future HIV vaccine interventions, promote related research in their communities, and mobilize others to the cause [12]. Similarly, in Tanzania parents, teachers and other community members were highly acceptable of vaccinating young girls against Human Papilloma Virus (HPV) with considerable support for a girl’s right to be vaccinated, as long as information was provided to the parents and communities [13].

Despite their moderate to high-level willingness to participate and higher risk of contracting HIV, young people might hesitate to participate in trials of HIV vaccines due to community attitudes. For example, in the Dominican Republic individuals considered to be at low risk of HIV infection feared that since the vaccine could be perceived as a cure for those already infected and that they would therefore be labeled ‘HIV positive’ [14]. They recommended that social support and counseling be employed to counteract potential negative social reactions [14]. Other barriers to participation involve concerns about possible influence on intimate relationships, negative experiences with health care providers, and concerns about confidentiality [15]. Thus, clear and appropriate information to community members would be one way to enhance participation in vaccine trials.

In connection with clinical trials, it is an ethical requirement that each participant agree to participate autonomously [6]; but this may not actually be possible in many low-income countries, where important decisions are taken by the family and/or community [16]. As such, it can be expected that Tanzanian youth would be influenced by their families when deciding whether or not to participate in a HIV vaccine trial, and, indeed 86.4% of young people in one study reported that they had someone close enough to influence such a decision on their part [17].

In light of the important contribution of community members to the recruitment and retention of volunteers in previous interventions designed to prevent HIV, too little is presently known about the views of community members in Tanzania and elsewhere on such participation by young people. Accordingly, the present investigation was designed to close this gap in our knowledge.

## Methods

### Study design and sampling

This study was conducted at the Infectious Diseases Clinic (IDC) in Ilala Municipality, Dar es Salaam, Tanzania, which provides youth-friendly services such as information concerning the adolescence and sex reproduction and contraceptive services, as well as HIV testing, care and treatment. This exploratory, qualitative study focused on perceptions of youth participation in trials of HIV vaccine among the friends/peers, parents, siblings and guardians (referred to collectively as “community members”) identified by youth in Dar es Salaam [17]. Study participants were recruited using purposeful snowball or chain sampling to obtain participants with the wealth of information required to answer research questions [18]. Potential participants were initially informed about the study by young people who felt close to them, and thereafter, we confirmed participation. All participants read and signed the informed consent prior to joining the study.

### Data collection and analysis

Data were collected in the form of focus group discussions (FGD) conducted between February and April 2013 to capture beliefs and other cultural factors that influence the feelings, attitudes, and behavior of individuals. FGDs can illuminate differences in the perspectives of different groups of individuals [19]. At the beginning of the FGDs, we asked for and received permission from the participants to record the discussions and in addition, both EAMT and TM took notes, which were used to pose follow up questions and incorporated into the transcripts.

In total seven FGDs were conducted, three with women and four with men, with 5–8 participants, each lasting 30–67 minutes. The FGD guide written in English and then translated into Kiswahili included socio-demographic information and three leading questions: 1. What impedes young people from participating in clinical trial? 2. How would you support such participation? and 3. What other facilitating factors might promote participation? The first author, (TM) and the second author, (EAMT) moderated sessions. We stopped after the seventh group discussion, when the lack of any new information, indicated that we had reached saturation [20].

A research assistant transcribed all of the FGD recordings verbatim. The first author, who is fluent in both English and Kiswahili, then translated these transcripts into English, reading all of them several times to make sure that she understood what the participants had said and, also listening to the audiotapes to make sure that all of the discussions were included in the transcripts. Employing content analysis [21], the results were expressed primarily as manifest and latent content. The views of community members deduced from sentences were incorporated into meaning units, which were then coded and abstracted, with the different codes written into the transcripts using different colors. The first (TM), and last authors, (AK), sorted the codes manually into categories, which were constructed, discussed, reassessed, and reviewed by TM, EAMT and AK, until agreement on three final themes was reached. Please see Table 1 for an example of this analytical process.

**Table 1:** An example of our qualitative content analysis.

Meaning unit	Code	Category	Theme
In connection with trials, people think they are being administered drugs to make them infertile, or infected with HIV to kill them	Side-effects, Infertility, Inefficiency of the HIV vaccine	Fear of perceived content of an HIV vaccine	Misconceptions concerning the HIV vaccine trial
There is a strong relationship between decisions made by a youth to participate in a trial and her/his parents’ support or lack thereof, we would be lying if we said a young person can decide independently of his/her parents since our youth have not been brought up that way	No authority in decision-making, a strained family relationship, disrespect to elders	Decision-making concerning participation	The covert power of community members to influence participation in the HIV vaccine trials

### Ethical considerations

The study was granted ethical approval by the Institutional Review Board at Muhimbili University of Health and Allied Sciences (MUHAS). The consent form described the aim of the study, the need for participation, and the right to withdraw, as well as the benefits and risks of participating. The first and second authors, (TM and EAMT) spoke to the participants who had additional questions before deciding to sign. All 44 participants provided their written consent before beginning the discussions. Each was reimbursed 7500TSH (equivalent to 5 US dollars) for transport to and from the study site (IDC).

## Results

Twenty six (59%) men and 18 (41%) women with a mean age of 27.4 years (range 18–47 years) participated. Seventeen (39%) had primary school and 27 (61%) had secondary school or higher education. Thirty eight (86%) participants were single (36 never married and 2 widowed) and 6 (14%) married. They included students, repairmen, social workers, and small business owners as well as some reporting no occupation at the time of the study. Three themes that emerged and relevant categories are presented below.

### 1. The covert power of community members to influence participation in the HIV vaccine trials

#### Decision-making concerning participation

Traditionally, decision making in any major social and health related event is viewed as a responsibility of elders rather than of young people in Tanzanian setting. The elders are expected to guide young people into making ‘right’ and ‘appropriate’ decision because of the perceived elderly wisdom and years of experience. Likewise with participation of young people in the HIV vaccine trials, the study participants, including the younger community members felt that young people lacked authority when it came to decision-making. This observation was expressed as:

“There is a strong relationship between decisions made by a youth to participate in a trial and her/his parents’ support or lack thereof. We would be lying if we said a young person can decide independently of his/her parents since our young people have not been brought up that way” (FGD 1, participant 3, male, age 28).

The participants perceived that it would be an act of disobedience by youth if youth decided to participate in the trials when families disagreed on. Consequently, it could lead to strained relationships within the family and community. The parents would feel angry and disrespected and cut off social support, and in the case of married young people, it could lead to marital conflicts. A participant expressed:

“It does not matter whether a young person is dependent or independent because there is always relationship between that person with a mother or a father or relatives. Therefore if s/he decides to volunteer in trial without blessing and then gets a problem, even if it is not related to vaccine, that person will not be able to get assistance from community because s/he will be rejected” (FGD 5, participant 3, male, age 24).

Most participants agreed preservation of habit of involving members of community in decision-making was an important matter for successful involvement of youth in the HIV vaccine trials. The participants reasoned youth needed harmonious and balanced social relations from different key players including spouses and parents in order for young people to stay focused, maintain psychological well being and for completion of trial schedule.

Others also expressed that sometimes some youth can decide to participate in the vaccine trials on their own without requiring parental permission as stated below:

“As long as a participant is sure and well informed that a trial would have no unpleasant effects, someone can participate without involving parents especially if s/he is 18 years and above” (FDG5, participant 2, male, age 25).

#### Community support for fear of new intervention

Impact of community support on participation of youth in the vaccine trials was central to narratives of the participants. The study participants said that community members would support youth because of ‘newness’ of the HIV vaccine trials in Tanzanian setting. The participants believed it was role of community members as parents, guardians, friends and peers to help young people facing perceived uncertainties, fears and unpleasant effects of an HIV vaccine on trial if such things happen as stated below:

“I will encourage young people to join and stay in trial. I will look for information about participants in previous HIV vaccine trials, this information will help me to ease their fear that they were not going to be the first one to participate in a vaccine trial.” (FGD 7, participant 5, male, age 26).

In addition, the participants spoke optimistically about supporting the HIV vaccination trials, even though the vaccine proposed had not been proven to be effective. The participants hoped that young people could be instrumental in achieving an effective HIV vaccine in the future. One participant stated:

“I would advise young people to participate because every new thing has a beginning even though we do not know that the vaccine would work effectively” (FGD 3, participant 3, female, age 25).

Furthermore, the participants’ support was raised in relation to HIV testing as a required step to qualify for participating in the vaccine trial, since the trial would recruit only HIV negative individuals. The participants shared experiences that in general youth did not like to be tested for HIV because of fear of social stigma attached to being HIV positive as illustrated here:

“We, parents need to be close to youth as most of them are afraid to test for HIV. Teach and encourage youth to take HIV test, as well as to participate in a vaccine trial. This distress of being found with HIV infection may prevent her/him from testing for HIV” (FGD2, participant 3, female, age 40).

A young participant further stressed the importance of preparing youth to test for HIV and deal with test result as illustrated below:

“It is important to make youth ready to be involved in HIV issues such as testing. Some youth may not want to be tested because youth fear that once it is known they live with HIV they will be stigmatized or the society will reject them” (FGD 5, participant 7, male, age 23).

### 2. Misconceptions concerning the HIV vaccine trial

#### Misunderstanding of sexual behavior of the trial participants

HIV vaccine clinical trials have an ethical obligation to ensure the volunteers are protected from acquiring any sexually transmitted infection, including HIV through provision of condoms and regular risk reduction counseling and assessment. The study participants perceived that young people would be asked to have unprotected sexual intercourse with HIV positive individuals as part of trial procedure in order to see whether or not the vaccine worked. However this belief was purely speculative, partly contributed by lack of knowledge about HIV vaccine and trial procedures among members of community.

One participant expressed that erroneous observation as:

“People are afraid of a trial because the participants need to have unprotected sexual intercourse with an HIV infected individual to see whether or not the vaccine works” (FGD 6, participant 3, male, age 24).

On other hand, there were participants who mistakenly expressed young people would be less likely to participate if young people were asked to abstain from sex as part of HIV preventive package during trial period. The participants mentioned that during adolescence and young adulthood, youth were very sexually active, and instruction like sex abstinence would limit participation of youth in the trials.

“It will be difficult for youth to participate if they are told not to have sex because most youth like sex” (FGD 7, participant 5, male, age 26).

#### Fear of perceived content of an HIV vaccine

In addition, the study participants had no prior knowledge on the composition of the HIV vaccine and how it worked. During the focus group discussions, it emerged that the participants believed young people might experience fear that the vaccine would contain an infectious HIV, which could infect young volunteers:

“We used to take our children for vaccination, whereby care providers used to inject some medication [vaccine] in body of the children. The same procedure, getting vaccine injection with HIV would be done for young people in the HIV vaccine trials; therefore young people would be afraid to join the trial” (FGD 4, participant 5, female, age 45).

In Tanzania, the ability to bear children is regarded as vital and sacred experience in perpetuation of future generations. However, many participants had the misunderstanding that the vaccine could contain substances that interfere with fertility thus reducing population growth. A young participant described:

“Most youth in my community do not trust people from the west because the westerners want to get hold of our wealth. The westerners introduce things like vaccines with an aim of reducing the native population’s ability to have children” (FGD 7, participant 3, male, age 23).

### 3. Potential structural impediments to following the trial routine

#### Stigma

During discussions stigma was shown to be an ever-present phenomenon in most HIV related activities despite existing HIV preventive programs in Tanzania setting. The participants stated that young people would be labeled as promiscuous and/or living with HIV because there was general perception in community that youth had sexual risk behaviors. This attitude would be further pronounced if young people would appear in interventions like the HIV vaccine trials as illustrated below:

“Young people may desire to participate in a trial but they think people would judge them as promiscuous since they volunteered for the trial” (FGD 3, participant 2, female, age 25).

Moreover, the participants assumed that stigma and doubt around participation in the HIV vaccine trials would be felt because of fear of isolation from community as expressed here:

“Once people from community know a young person has been vaccinated [becomes a trial participant], they will think that person lives with HIV, as a result s/he may be isolated by community” (FGD 1, participant 3, male, age 28).

#### Unemployment and poverty

The participants stated that most Tanzanian youth have hard lives, with low socioeconomic status. The participants expressed that most youth, despite being at legal age of 18 years and above, depended socially and economically on their parents. The participants said that unemployed youth might be too busy looking for jobs to participate in vaccine trials:

“Most of time youth are busy, looking for means to earn living. It would be difficult for them to leave daily activities to attend to meetings for discussing vaccine issues” (FGD 5, participant 7, male, age 23).

#### Substance abuse

The participants were concerned that substance abuse by some young people would not only impede the trial but also expose them to HIV. The participants said most youth were victims of drug abuse. Using drugs had made youth hostile and violent as well as acquiring sexual risk behaviors. Due to such misbehaviors, the participants felt such young people would be unfit to join the vaccine trials as expressed below:

“Most youth are involved in risky sexual activities influenced by injectable drugs, peer pressure, alcoholism, and smoking weed. For example, if a young woman receives [a vaccine], then drinks excessively, then end up having unprotected sex, such volunteer would not be able to follow vaccine trial to the end because of such behavior” (FGD 1, participant 3, male, age 28).

## Discussion

This study highlights the important role of community members in decision-making concerning participation in HIV vaccine trials by Tanzanian youth who are, and sheds light on factors that may impede or facilitate such participation by young people in Dar es Salaam. Community members appear to provide key support to most Tanzanian youth at or above the legal age of adulthood. This study finding demonstrates that preventive biomedical interventions require support from, acceptance by, and collaboration with community since potential young participants in HIV interventions depend on members of their community for social, moral and sometimes even economic support. In agreement, another study conducted by one of the authors here revealed 84.6% of young participants had someone significant who influenced their decisions on important social and medical matters [17].

Furthermore, these current findings reflect accepted norms and traditions that urge young people to listen to and follow the advice given by members of their community, especially elderly members, concerning important decisions in life in Tanzanian setting. For instance, Nigerian women seek permission from their spouses before consenting to participate in research [16]. In addition, women, as mothers and as partners, are likely to exert considerable influence on the acceptability of male circumcision as an intervention to control the spread of HIV in many African settings, and any such effort would be more successful if it appeals to both women and men [22]. Therefore, when designing a HIV vaccine trial involving young people the views of community members should be considered in order to integrate this intervention into the complex local pre-existing customs, norms, beliefs and practices. This will, in turn, improve recruitment and retention [23], as well as reduce discrimination and disharmony in the family and/or community.

In addition, we identified a desire among community members to encourage young people in Tanzania to play an important part in development of an effective HIV vaccine. Such participation in vaccine trials is novel in Tanzania and our participants expressed a sense of collective responsibility for the welfare of any young person, stating that they would offer moral support to face fears, stigma and uncertainties during trial procedures. This shows commitment and communal duty of community members to safeguard good health of youth.This finding supports the beliefs that any program’s success depends as much on the wider community context into which it is implemented as on the technical details [24]. Similarly, in Atlanta, Georgia, USA; community-level support and medical and treatment programs promoted participation in HIV vaccine research [25].

The importance of accurately explaining the content of a trial vaccine is another important outcome here. Most of the participants’ misconceptions on trial procedures and content of vaccine were unfounded. It is an ethical obligation for clinical trial teams to ensure the trial volunteers are counseled to practice safer sex behaviors. Counseling reinforces the need to avoid both sexually transmitted infections, including HIV, and pregnancy. In most trials volunteers receive condoms as part of risk-reduction counseling [6]. Our study participants’ beliefs that the vaccine could cause side effects seem to have contributed to their fear and hesitancy about encouraging young people to participate in the trials, beliefs that may, at least in part, be due to a lack of appropriate information. Identical findings have been made in South India, whereby frontline providers of health services showed a lack of knowledge concerning clinical trials. There, the participants expressed concerns that an HIV vaccine might promote unprotected sexual practices as well as sexual activity in general. Others feared losing the trust of community in the event of harmful side effects [26].

Clearly, it is important to provide appropriate information, not only to potential trial participants make a decision also to allow community members to weigh the advantages and disadvantages of trial participation when advising young people on this matter.

Our present results are in agreement with previous studies on the influence of misunderstandings on participation in HIV vaccine trial. Our participants felt that young people might shy away from volunteering in the vaccine trials due to misinformation, such as that the vaccine could lead to infertility, encourage participants to have unprotected sexual intercourse with HIV infected individuals or, even worse, infect them with HIV. Our participants were echoing misconceptions by young people, but also airing their own suspicions surrounding HIV vaccine research in general. Parents in South Africa were reported to misunderstand the potential for harm from a HIV vaccine, microbicides, and a pre-exposure prophylaxis trial, as a result refusing to let their children participate [27]. In addition, community members influence retention negatively, even when informed consent has been obtained. For example, in Tanzania, police officers declined to participate in a HIV vaccination trial because a significant number of others mistrusted the vaccine [28].

Unfortunately, such misconceptions exert a detrimental impact not only on trial participation, but also on the acceptability and usage of newly licensed vaccines. Thus there was hesitancy to use influenza vaccines in America due to unfounded fears regarding transmissibility, safety, and efficacy [29]. Similarly, a meta- analysis of attitudes about and usage of the human papilloma virus (HPV) vaccine revealed that most fears were based on myth rather than reality. These vaccines are safe and do not increase sexual activity among young people, despite the beliefs of some Americans to the contrary [30].

Finally, our results indicate that common structural problems such as poverty and substance abuse constitute barriers to recruitment to and retention in HIV vaccination trials. Young people living in poverty without opportunity are easy targets for the global drug trade [31]. Moreover, drug use is linked to instability, violence, difficulties working, work overload, neglect and abuse [32], making it difficult for such youth to be recruited and retained in HIV vaccine trials.

Stigma was cited as another barrier keeping youth from participating in HIV vaccine trials. Our participants felt that young people are afraid of being discriminated against, rejected, and/or seen as promiscuous if they do participate in such trials. In South Africa respondents thought that this stigma originates in the association between HIV and sex [32 33]. Such stigma may continue even after termination of an HIV vaccine trial if adequate information concerning post vaccination effects such as vaccine induced sero-positivity (VISP) is not provided appropriately to members of community. VISP can lead to disruption of personal relationships; difficulties in finding or keeping employment; difficulties in obtaining insurance; impediments to travel; the inability to enlist into the military; the inability to donate blood, sperm, and organs; and inappropriate medical treatment [34]. Altogether, the challenge present by this study involves providing useful, practical and culturally sensitive information about participation in HIV vaccine trials designed to alleviate fears, worries and hesitancy among members of community.

### Methodological considerations

Since we explored, in detail, the views and opinions only of people close to youth who had visited the IDC, the views of our participants cannot be considered representative of all such individuals in Tanzania. In addition, our sampling could be biased, since young people who had expressed a willingness to participate in a hypothetical vaccination trial referred our participants to us. Our results may have been different if we had sampled, e.g., through youth who were unwilling to participate in the vaccination trial. Nevertheless, we believe the information we have obtained is of considerable value in connection with designing future HIV vaccine trials in Tanzania.

Furthermore, here we did not assess our participants’ understanding of HIV and HIV vaccines, information that could have provided more insight into responses concerning whether decision to support the participation of youth in HIV vaccine trials or not. However, we did brief the participants about the meaning and intention of HIV vaccine trials, with examples, before conducting the FGDs.

In summary, our present findings show that community members are an essential element in the decision to participate in an HIV vaccine trial and would be willing to support such trials. The study participants pointed out certain misconceptions and structural barriers associated with this decision-making that can and should be addressed. Consequently, we conclude that community members can play an instrumental role in the recruitment and retention of young people to HIV vaccination trials.

### Implications

Our results demonstrate a need to involve and thoroughly inform individuals close to young people about the vaccine trial so that community members understand the aims, process, and requirements for participation. Moreover, any upcoming trial involving young people should provide youth-specific educational material that addresses issues such as stigma and substance abuse to help maximize retention in the trial.
